# Lipodystrophy With an Uncommon Clinical Presentation in a Patient With Insulin-Requiring Type 2 Diabetes Mellitus

**DOI:** 10.7759/cureus.73835

**Published:** 2024-11-17

**Authors:** Chaima Jemai, Achwak Mehrez, Nesrine Dhieb, Yosra Htira, Faika Ben Mami

**Affiliations:** 1 Department C, National Institute of Nutrition of Tunis, Tunis, Tunisia; 2 Department C, National Institute of Nutrition of Tunis, Tunis, TUN

**Keywords:** insulin therapy errors, lipodystrophy, patient safety-based medical education, recurrent hypoglycemia, type 2 diabetes mellitus

## Abstract

We report the case of a patient with type 2 diabetes mellitus (T2DM) on insulin therapy with a history of recurrent and severe hypoglycemia related to lipodystrophy with an uncommon clinical presentation. This was the case of a 67-year-old female with type 2 diabetes hospitalized for the exploration and management of severe and recurrent hypoglycemia. Her diabetes has been evolving since the age of 40 years and was complicated by minimal retinopathy. She was on premixed human insulin, administered through an insulin syringe for the last 17 years. She presented a history of well-controlled diabetes until five months back when she started to show a fluctuating blood glucose concentration, with episodes of unpredictable hypoglycemia occurring at variable times, with values inferior to 0.3 g/l associated with neurological features. Clinical examination revealed a swelling localized in the hypogastric region of the abdomen. It was painless, firm, not fixed to the underlying plans, without local inflammatory signs, and had appeared in the patient's preferred insulin injection site. Thus, we retained the diagnosis of insulin-induced lipohypertrophy. The patient has reported reusing needles for up to one week for economic reasons, and not frequently rotating insulin injection sites. The patient found a less painful injection in the lipohypertrophic area, and she continued to inject insulin into that zone, leading to its progressive enlargement. Therapeutic management consisted of switching the patient to insulin analogs and resuming education concerning the correct injection techniques. The insulin injection technique continues to be suboptimal in many insulin-treated patients, and our case emphasizes the need for improved awareness and education.

## Introduction

Lipodystrophy is a common complication of a subcutaneous insulin injection in patients with diabetes mellitus, with prevalence estimated at 41.8% [1]. It can be defined as a tumor whose development is stimulated by the local injection of insulin, a fat-development-promoting hormone. This is a problem worth discussing, given its aesthetic and metabolic burden: this benign swelling might affect insulin absorption, resulting in the erratic absorption of injected insulin, leading to extreme glycemic variability with a negative impact on glycemic control [2,3]. Hypoglycemia is the most challenging feature of glycemic variability related to lipodystrophies. Lipodystrophies are unpredictable, could be severe, might be life-threatening, and negatively impact life quality with even psychiatric problems.

Precise recommendations are lacking, and managing lipodystrophies remains challenging. We aim to enrich the field by reporting the case of our patient with type 2 diabetes on insulin therapy who had lipodystrophy with an uncommon clinical presentation.

## Case presentation

We report the case of a 67-year-old female with type 2 diabetes female hospitalized at Department C of Diabetology in the National Institute of Nutrition of Tunis, for exploration and management of severe and recurrent hypoglycemia. She had a medical history of hypertension and dyslipidemia. Her diabetes has been evolving since the age of 40 years and is complicated by minimal retinopathy. She was on premixed human insulin, administered through an insulin syringe, for the last 17 years.

She presented a history of well-controlled diabetes (Hba1c=6.3%) until five months back when she started to show a fluctuating blood glucose concentration, with episodes of unpredictable hypoglycemia occurring in variable times, with values below 0.3 g/l associated with neurological features (agitation and aphasia). She reported no other symptoms. Her weight status was normal, with a 20 kg/m² BMI. There was no story of involuntary weight loss.

She did not report excessive physical activity or changes in dietary intake, and she injected insulin in the hypogastric region of the abdomen.

**Figure 1:**
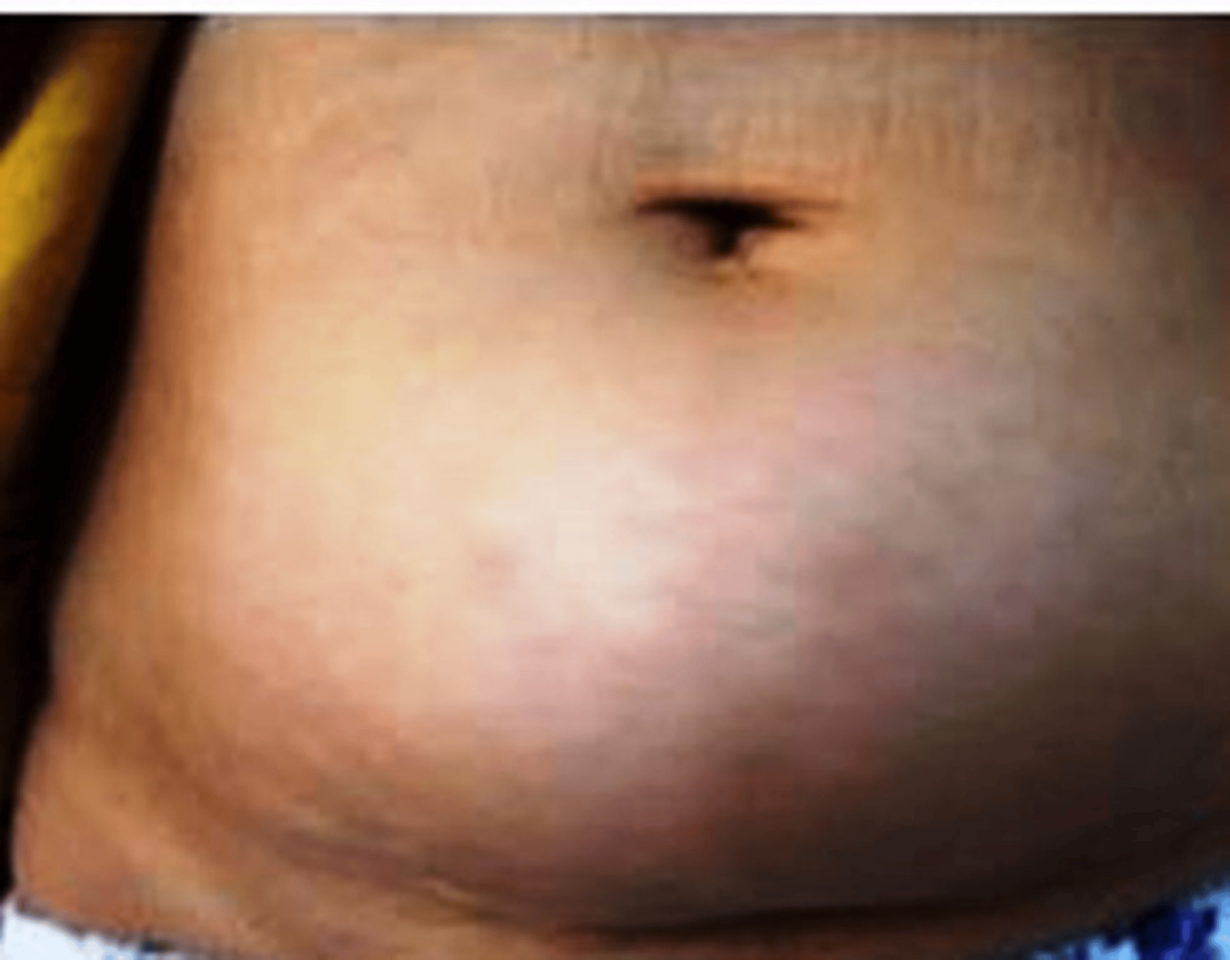
Monster insulin-induced lipodystrophy One major lipohypertrophy swelling localized in the hypogastric region below the navel. It was markedly protruding from the cutaneous plane and was firm and painless on palpation.

The clinical investigation of this unusual injection site, revealed a swelling, as shown in Figure 1. It was painless, firm, not fixed to the underlying plans, without local inflammatory signs.

On reviewing the injection technique, the patient has reported reusing needles for up to one week for economic reasons and not frequently rotating insulin injection sites. She found a less painful injection in this lipohypertrophic area, and she continued to inject insulin into it, leading to its progressive enlargement. Thus we retained the diagnosis of insulin-induced lipohypertrophy.

The biological test revealed glycated hemoglobin (HbA1c) of 8.7%, normal renal, hepatic, and thyroid parameters, and normal basal cortisolemia. The lab investigation results are reported in Table 1. Thus, organic causes have been ruled out, and lipohypertrophy was retained as the cause of hypoglycemia in our patient.

**Table 1:** Laboratory investigation results HbA1C: glycated hemoglobin; CKD-Epi: Chronic Kidney Disease Epidemiology Collaboration; AST: aspartate aminotransferase; ALT: alanine aminotransferase; TSH: thyroid-stimulating hormone

	Patient's value	Normal range
Fasting blood glucose (mmol/L)	4,8	4.1-6.1
HbA1C (%)	6,1	4.5-6
Creatinemia (umol/L)	62	135-145
Creatinine clearance (ml/mn) (CKD-Epi formula)	89.44	
AST (IU/L)	28	<45
ALT (IU/L)	21	<40
Thyroid-stimulating hormone (TSH) (IU/L)	2,4	0.34-5.6
Baseline cortisolemia (nmol/L)	237	138-500

The therapeutic management consisted of switching the patient to insulin analogs and resuming the education concerning the correct injection techniques; she was advised not to inject insulin into the swelling anymore, to rotate injection sites, and to minimize the reuse of needles.

Surgical resection of the lipodystrophy, for aesthetic purposes, was proposed but refused by the patient. We plan to follow up with our patient to monitor the glycemic balance and the evolution of lipodystrophy.

## Discussion

Lipodystrophy is a common adverse effect of insulin subcutaneous injections, especially in patients with type 1 diabetes mellitus, with a prevalence estimated at 41.8% according to a recent meta-analysis [1]. It is related to immunologically mediated changes in the subcutaneous fatty tissue [2,3]. Injection into lipodystrophied sites results in an abnormal absorption of insulin, leading to glycemic instability and uncontrolled diabetes [4].

Repetitive needle use for economic or personal convenience, number of daily injections, not rotating injection sites, depth of insulin application and injection area size, and long duration of insulin therapy are the most incriminated factors [5-8]. Likewise, several clinical features, such as younger age, lower BMI, and higher daily insulin dose, have been demonstrated as factors associated with increased lipodystrophy risk in other studies [9-11].

Our case constitutes an extreme case with a monster lipodystrophy developing in an unusual location and resulting in erratic glycemic variability and severe unpredictable hypoglycemia. Thus, both vital and esthetic prognoses are engaged, and liposuction may be required to manage this complication [12,13].

It seems that despite education, patients may keep having educational deficiencies and bad habits are difficult to rule out. This case emphasizes that patients and caregivers should have enough information about lipodystrophies.

Periodic resumption of therapeutic education coupled with regular examination of injection sites is recommended in all insulin-treated patients. Inspection and palpation should be associated with optimal screening. Thereby, a meticulous clinical examination could spare further invasive examinations.

## Conclusions

Diabetes mellitus is an expanding health problem worldwide. Insulin therapy continues to be a leading therapeutic of this metabolic pathology, largely prescribed because of its low cost and high efficacy. However, it could lead to some serious complications.

The case of our patient highlights one of those complications, lipodystrophies. Managing related hypoglycemia was challenging. Moreover, advanced age exposes the patient to an additional risk of related hypoglycemia-cardiovascular acute events. These findings implicate an undoubted need for structured recommendations. We propose that these recommendations particularly highlight how to rule out bad consolidated habits especially restricted injection sites, as it is the most incriminated factor in the development of lipodystrophies.
